# Why COVID-19 is Less Severe in Pediatric Patients?

**DOI:** 10.4314/ejhs.v30i4.1

**Published:** 2020-07-01

**Authors:** Abraham Haileamlak

SARS-CoV-2 is one of seven coronaviruses known to infect humans. Other than SARS-CoV-2, the other coronaviruses causing SARS and MERS are deadly, the rest are relatively benign causing only common colds in the vast majority of cases. SARS-CoV-2 indiscriminately infecting people with varying severity across geographies, genders, and occupations. Despite this indiscriminate attack, experiences and studies indicate that children are mildly affected in comparison to adults, representing approximately 5% of cases and less than 1% of admissions to hospital (1, 2). The proportion of severe and critical cases was 10.6%, 7.3%, 4.2%, 4.1%, and 3.0% for the age group of .1, 1–5, 6–10, 11–15 and >15 years, respectively (1). SARS and MERS showed similar pater where kids are largely spared from severe diseases. Swine flu (H1N1) virus, responsible for the flu pandemic of 2009 and 2010 preferentially had tummy symptoms on children.

It became mysterious why children are less sick after being infected where more than 90 percent of pediatric cases presenting as moderate, mild, or without symptoms entirely. “Why most of the children's COVID-19 cases were less severe than adults' cases is puzzling. Scientists and clinicians need to learn more about this virus and the immune response against it at different age groups. Untying the mystery why SARS-CoV-2 is less severe in children could help to design new ways to combat the spread of the disease.

Different literatures forwarded the following ideas to explain why children develop mild COVID-19 disease.

1. **Vulnerability to ARDS:** though small number of cases pass through all phases, COVID-19 disease is having three distinct phases namely the viral phase, the pulmonary phase, and the hyperinflammatory phase. It is during the hyperinflammatory phase cases are likely to develop severe complications like acute respiratory distress syndrome (ARDS). However, studies showed that children are not less prone to developing ARDS during respiratory tract infections; in fact, during the H1N1 flu pandemic, being under the age of one year was a significant risk factor for developing a severe form of ARDS (3,4). Therefore, this argument cannot hold true.
2. **The immune system's delicate balance:** One of the reasons for the milder COVID-19 disease presentation in children compared to adult may be due to a qualitatively different response to the SARS-CoV2 virus (5). With increasing age, children's immune systems may reach a sort of just-right status, growing strong enough to keep an infection in check without overreacting.
3. **Prior Exposure to other similar viruses:** children are exposed to various similar viral infection while they are in kindergarten and school. Prior exposure to other milder viruses may contribute for children's less severe COVID-19 disease. It is shown that disease development and subsequent severity is dose dependent as it is shown in other studies (6). The presence of other viruses in the airways of children could limit the growth of SARS-CoV2 by direct virus-to-virus interactions and competition (7).
4. **Expression level of Binding Proteins:** Similar to that of SARS-CoV-1, SARSCoV-2 establishes infection by glomming on to a protein called angiotensinconverting enzyme (ACE)2 which is found on the surfaces of cells throughout the body (8). Differences in the expression levels of the ACE-2 receptor between children and adults could contribute for the less severe disease in children. Some researchers have hypothesized that kids' lung cells could make fewer―or perhaps even differently shaped-ACE2 proteins. This is an example of the fact that the immune systems of children and adults are different, both with respect to their composition and functional responsiveness (9).

To note, although children tend to have mild forms of COVID-19, protective measures should be taken to prevent them from becoming infected. This is important for mitigating the pandemic, as not only can children transmit viruses even when presenting mild forms of the disease, but they have been shown to harbour large amounts of the virus even without showing symptoms (10). It is important to remember that viruses can persist in faeces long after they are gone in nasopharyngeal secretions.

Understanding the milder COVID-19 disease in children will provide important information about the disease. It may also suggest important protective mechanisms and targets for future therapies.
